# Perceptions, Vulnerability and Adaptation Strategies for Mitigating Climate Change Effects among Small Livestock Herders in Punjab, Pakistan

**DOI:** 10.3390/ijerph182010771

**Published:** 2021-10-14

**Authors:** Muhammad Faisal, Azhar Abbas, Yi Cai, Abdelrahman Ali, Muhammad Amir Shahzad, Shoaib Akhtar, Muhammad Haseeb Raza, Muhammad Arslan Ajmal, Chunping Xia, Syed Abdul Sattar, Zahira Batool

**Affiliations:** 1 College of Economics and Management, Huazhong Agricultural University, Wuhan 430070, China; faisalgurmani@gmail.com (M.F.); aaa31@fayoum.edu.eg (A.A.); sivia.amir@gmail.com (M.A.S.); xcp@mail.hzau.edu.cn (C.X.); 2 Institute of Agricultural and Resource Economics, University of Agriculture Faisalabad, Faisalabad 38000, Pakistan; azhar.abbas@uaf.edu.pk; 3 Research Center for Green Development of Agriculture, Digital Countryside Research Institute, College of Economics and Management, South China Agricultural University, Guangzhou 510640, China; 4 Department of Agricultural Economics, Fayoum University, Fayoum 63514, Egypt; 5 Centre of Excellence for Olive Research and Training (CEFORT), Barani Agricultural Research Institute, Chakwal 48800, Pakistan; shoaibakhtar1799@gmail.com; 6 Institute of Business Management Sciences, University of Agriculture Faisalabad, Faisalabad 38000, Pakistan; haseebrizvi00@yahoo.com; 7 Department of Economics, Government College University, Faisalabad 38000, Pakistan; arslan.gogo7@gmail.com; 8 Agriculture Research Institute, District Kharan, Balochistan 94100, Pakistan; syedabdulsattar1975@gmail.com; 9 Department of Sociology, Government College University, Faisalabad 38000, Pakistan; batoolazam@hotmail.com

**Keywords:** perception, vulnerability, livestock herders

## Abstract

Pakistan is an agrarian nation that is among the most vulnerable countries to climatic variations. Around 20% of its GDP is produced by agriculture, and livestock-related production contributes more than half of this value. However, few empirical studies have been conducted to determine the vulnerability and knowledge of livestock herders, and particularly the smaller herders. Comprehending individual perceptions of and vulnerabilities to climate change (CC) will enable effective formulation of CC mitigation strategies. This study intended to explore individual perceptions of and vulnerabilities to CC based on a primary dataset of 405 small livestock herders from three agro-ecological zones of Punjab. The results showed that livestock herders’ perceptions about temperature and rainfall variations/patterns coincide with the meteorological information of the study locations. The vulnerability indicators show that Dera Ghazi Khan district is more vulnerable than the other two zones because of high exposure and sensitivity to CC, and lower adaptive capacity. However, all zones experience regular livelihood risks due to livestock diseases and deaths resulting from extreme climatic conditions, lower economic status, and constrained institutional and human resource capabilities, thus leading to increased vulnerability. The results indicate that low-cost local approaches are needed, such as provision of improved veterinary services, increased availability of basic equipment, small-scale infrastructure projects, and reinforcement of informal social safety nets. These measures would support cost-effective and sustainable decisions to enable subsistence livestock herders to adopt climate smart practices.

## 1. Introduction

In the 20th century, climate change (CC) has posed significant challenges for nations and the global community, in addition to posing threats for future generations [1,2,3]. Climatic variations, in the form of erratic rainfall, intermittent droughts, deadly cyclones, and heat waves, pose threats to all sectors of the economy and walks of life, both marine and land-based [4]. Due to its high dependence on natural resources, such as water, temperature, light, soil, and oxygen, and vulnerability to events that may result from any natural imbalance, the agriculture sector is one of the most sensitive to CC, thus threatening millions of subsistence farmers who heavily rely on the sector’s performance [5,6]. In developing countries, the level of vulnerability of small farmers to CC is further intensified because of their low adoptive capacity, poor institutional support, and the temporary nature of resilience-enhancing infrastructure [7,8]. The current risk to households’ well-being and food security is higher in these countries because smallholders’ livelihoods are more exposed to CC [9,10,11]. It is expected that CC will affect the occurrence of diseases, increase the severity and frequency of floods and droughts, increase the probability of crop failure, decrease yields, and increase livestock mortality [6,11,12,13]. Considering the close association between individuals’ income and agricultural production [14], the negative impact of CC on livestock may increase the vulnerability of small livestock herders. However, the degree of vulnerability of a location, system, or household is determined by socioeconomic and environmental factors [15].

Understanding the vulnerability of smallholders’ livelihood to climatic extremes against the background of broader transformational shifts in social and regional dynamics, in addition to the multidimensional perspective, has almost become a normative priority in recent years [16], although climate-related questions are debatable for a number of reasons. For instance, a relevant question is whether individuals are capable of noticing or monitoring CC. A second question relates to how individuals respond to climate-related (formal or informal) investigations, given the fact that CC is a long-term phenomenon and individuals have only short-term experiences. A third question relates to an individual’s ability to detect changes in atmospheric conditions based only on past memories, given atmospheric change is a slow process that can only be detected with meteorological devices [17,18]. Despite these practical issues, previous researchers [19,20,21,22,23] have tried to explain how individuals comprehend and interpret CC.

The main explanation for individuals’ poor comprehension, lack of concern, and limited evaluation regarding CC stems from inadequate and scantly available information [17], and a lack of relevant and timely data from relevant authorities. This lack undermines the ability to effectively adapt [18,24]. To identify CC, individuals must know the significance of CC perception and the adoption of mitigation measures. However, perception of CC is a personal assessment [25] that comprises an individual’s understanding, which in turn motivates actions with respect to CC incidence and severity [26]. Thus, an individual must perceive CC before responding to it, and this perception needs to be linked with actual CC for effective adaptation measures. However, it is expected that some—if not all—farmers may not be well placed to detect the abrupt changes resulting from environmental variation [27].

Due to the current pace of CC and its associated impacts, nations must consider CC seriously [28]. Various approaches are taken by individuals and societies to safeguard themselves against the effects of the weather. The extant literature has examined the multidimensional perspectives of CC with an emphasis on risk perception, potential barriers, impacts, adoption intensions, and adaptations in different areas [6,11,29,30,31,32,33,34,35,36]. Ahmad and Ma [16] highlight individual perceptions of CC extreme events, and compare these with meteorological data on temperature and rainfall. Hasan and Kumar [17] report that Bangali (Kalapara) rural people’s observations of extreme climatic events with regard to CC perceptions are generally consistent with the scientific evidence. There is also ample evidence on the potential role of region-wise vulnerability assessments to assist in developing national strategies for CC adaptation and facilitating the development of the adaptive capacity of vulnerable communities [8]. To the best of our understanding, few studies have considered CC vulnerability pertaining to Pakistan [1,16,37]. Surprisingly, no study has been performed in Pakistan in the context of region-wise assessment of livestock herders’ perceptions and vulnerability to CC, including a comparison to meteorological data. Comprehending individuals’ perceptions of CC and vulnerability would be beneficial for the formulation of effective adaptation strategies [5,8,14] that would ultimately help achieve sustained social and economic development among nations and regions [6,11]. Moreover, CC adaptation measures have numerous benefits [11]. Therefore, to expand the adoption and promotion of CC measures, it is important to explore the determinants of adoption.

Against this background, this study aimed to explore the vulnerability of small livestock herders from a multifaceted and multidimensional perspective, with the intention of determining the perceptions of CC of respondents’ in three agro-ecological zones of Punjab, Pakistan. The novelty of this study lies in its contribution to a deeper understanding of livestock herders’ perceptions about CC indicators, namely, rainfall, temperature, droughts, flood, and livestock diseases, by assessing their consistency with meteorological information. Such an evidence-based comparison is generally rare in the case of Pakistan and other parts of the world. Based on the vulnerability-level outcomes from the three studied zones, the study suggests suitable policy options that may help the public and private sectors to effectively plan for effective mitigation of the harmful effects related to CC.

### Vulnerability Assessment

Vulnerability assessment is a complex and a multidimensional concept. Its level varies across temporal and spatial scales while heavily depending on demographic, socioeconomic, geographic, cultural, institutional, governance and environmental factors [38,39,40,41]. It is considered in various dimensions according to its requirements [42,43]. There are numerous approaches and interpretations of vulnerability [41,44,45,46], although there is little consensus about its definition [47,48,49]. Brooks et al. [50] define vulnerability as a degree of exposure/risk and incapability to fight climatic variations. Regardless of the different definitions of vulnerability, the most accepted and comprehensive definition provided by the Intergovernmental Panel on Climate Change (IPCC) [51] is: “The degree to which a system, location, or household is susceptible to, or incapable to cope with adverse effects of climate variability and extremes. It is a function of character, magnitude, and rate of climatic deviations to which a system, location, or household is exposed, sensitive, and its adaptive capacity.” This interpretation is also typically acknowledged by the academic community [52]. Thus, three elements of vulnerability are consistently considered in the literature: first, exposure to climatic extremes; second, sensitivity to those climatic extremes; and third, the adaptive capacity to cope or recover from climatic extremes [12,40,53].

Considering vulnerability dimensions, exposure is the extent or level to which a system is exposed to major climatic deviations. Sensitivity means the degree to which a system is affected (directly or indirectly) by climatic stimuli, either positively or negatively. Finally, adaptive capacity means the capability of a system to effectively respond to climatic variations, and may involve adjustments in behavior, resources, and technologies [8]. The most extensively-used approach for CC vulnerability assessment is based on the framework suggested by the IPCC. This offers a suitable mechanism to recognize the causes of environmental disaster [54] and proposes appropriate adaptation measures to mitigate its adverse impacts [55,56]. The vulnerability assessment approach can be executed at different scales, such as the household or individual level, community level, regional, or country level [57,58,59]. However, as noted by Pearson et al. [60], there are numerous concepts and means of assessing vulnerability that occasionally overlap with each other. Nevertheless, the major aim of the vulnerability assessment is to focus the development of appropriate policies that may increase sectors’ resilience against CC [50,57,61].

## 2. Materials and Methods

### 2.1. Study Area, Sampling and Data Collection Method

**Figure 1:**
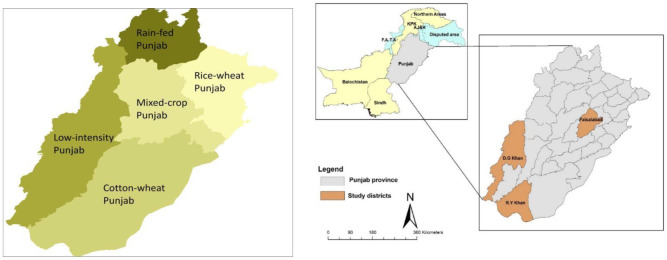
Map categorizing the agro-ecological zones of Punjab and the study area.

**Figure 2:**
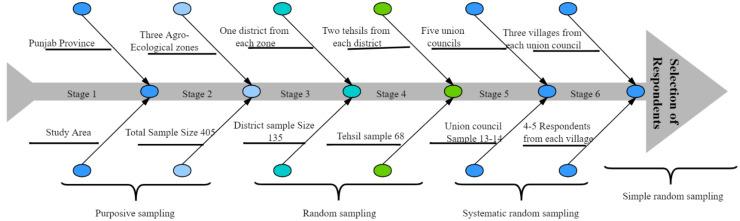
Sampling strategy.

The target population for the current investigation consisted of small livestock herders from three agro-ecological zones (Dera Ghazi Khan (DGK) from the low intensity zone, Rahim Yar khan (RYK) from the cotton-wheat zone, and Faisalabad (FSD) from the mixed cropping zone) of Punjab province in Pakistan (Figure 1). A multistage sampling strategy was used for data collection. Thirty villages were chosen through a field survey conducted in each agro-ecological zone, and 4–5 households were then randomly selected from each village (Figure 2). In total, field-level primary data were collected from 405 small livestock herders using a pre-tested questionnaire. Responses were verified from key informant interviews before final field observations. The questionnaire was primarily constructed in accordance with the literature [62,63]. A structured questionnaire for data collection was divided into different sections, including general information, household characteristics (socio-economic characteristics), farm characteristics, institutional characteristics, accessibility and availability of resources, assets (livestock and household assets), household income (off/on-farm income), and household perception of climate (risk perception, risk experience, and impacts) to assess exposure to CC, adaptive capacity at the household level, and intentions to adopt practices in response to CC. The indicators used in the current study were primarily based on authors’ own understanding of the study location, in addition to peers’ knowledge and the published literature [4,5,6,8,11,16].

Data collection was undertaken between January and June 2019 by the trained interviewers through face-to-face questioning in the local (*Saraiki* and *Punjabi*) languages. Because the questionnaire language was English, this facilitated the interpretation of the message by the respondents, whose literacy rate was low. On average, interviews took 30–40 min. A confidential protocol was followed related to identification of respondents and the village information, which were confined solely to the serial number of the questionnaire. For the current investigation, meteorological data were obtained from the Punjab province’s meteorological department for the period 2010–2019 related to the selected three agro-ecological zones. Quantitative (socio-demographic and economic characteristics, etc.) and qualitative (risk perception of frequency of events/variations over the past 10 years, etc.) data were used to analyze the survey information. Statistical Package for Social Science (SPSS) 24.0 and an MS-Excel work sheet were used for data analysis.

### 2.2. Climate Change Risk Perception Index

In this study, we used the climate change risk perception index (CCRPI) for the calculation of livestock herders’ perceptions of climatic events/variations that occurred during the past 10 years but rarely occurred previously [64,65]. A five-point Likert scale was used to collect risk perception data, from the past ten years (2010–2019), of climatic events/variations from 405 respondents. The scale ranges from very low/no perception to very high perception. For the calculation of CCRPI, we assigned a specific value to each perception scale: 4 for very high perception, 3 for high perception, 2 for medium perception, 1 for low perception, and 0 for very low/zero perception. Respondents’ evaluations of each climatic event were obtained by interview and recorded as frequencies. The following equation was used to estimate the climate change risk perception score (CCRPS):

$$CCRPS=CCRP_{vh}*4+CCRP_{h}*3+CCRP_{m}*2+CCRP_{l}*1+CCRP_{vl/0}*0$$

where $CCRP_{vh}$ is the frequency of respondents having very high perception, $CCRP_{h}$ is the frequency of respondents having high perception, $CCRP_{m}$ is the frequency of respondents having medium perception, $CCRP_{l}$ is the frequency of respondents having low perception, and $CCRP_{vl/0}$ is the frequency of respondents having very low/zero perception. Moreover, CCRPS for any climatic events/variation ranged from lower boundary to higher boundary, i.e., from 0 to 1620, respectively. For further interpretation, we transformed CCRPS into a standardized index. The following equation was used for standardization:

$$\begin{array}{l} Standardized\;climate\;change\;risk\;perception\;index\;\left(SCCRPI\right)=\\ Total\;CCRPS/Maximum\;boundary\;value \end{array}$$

The SCCRPI values ranged from 0 (minimum level of risk perceived by livestock herders) to 100 (maximum level of risk perceived). We then ranked this score.

### 2.3. Vulnerability Index

For the estimation of vulnerability, the IPCC framework was used [38]. This study adopted the index-based method used by Dendir and Simane [4] to calculate small livestock herders’ vulnerability levels. By the following vulnerability assessment module, relevant indicators were calculated from major and sub–components of a specific dimension/domain. Each major component included varying numbers of sub-components. All the indicators were normalized using a balanced weighted average approach, thus assuming that all indicators contributed equally to the overall index according to the functional relationship with vulnerability [4,14]. To standardize the indicators, Equation (1) was used as:

(1) $$index_{xv}=\frac{X_{v}-X_{\min}}{X_{\max}-X_{\min}}$$

where $X_{\min}$, $X_{\max}$, and $X_{v}$ are, respectively, the minimum, maximum, and actual value of specific indicator for a particular household, across all households [5]. For the calculation of each major component value, each indicator of sub-components was standardized and then averaged using Equation (2):

(2) $$M_{v}=\frac{\sum_{i=1}^{n}IndexX_{vi}}{n}$$

where $M_{v}$ denotes one of the 11 major components of the vulnerability, namely, extreme events, climatic variables, food and health, land and livestock, livelihood, belonging to the vulnerable group, adaptation efficacy, self-efficacy, economic capability, human resource capability, and institutional capability; the sub-components are represented by the index, where the index is denoted by i and n denotes the number of sub-components for each major component. Once values for each of the 11 major components for three agro-ecological zones were calculated, they were then averaged using Equation (3):

(3) $$LVI_{v}=\frac{\sum_{i=1}^{11}W_{mi}M_{vi}}{\sum_{i=1}^{11}W_{mi}}$$

where $W_{mi}$ is the weight of each major component. The LVI is scaled from 0 (least vulnerable) to 0.7 (most vulnerable). Because the IPCC framework was used for the estimation of the contributing factors (exposure, sensitivity, and adoptive capacity) of vulnerability [66], we placed exposure under extreme events and climatic variables. Sensitivity is defined as the food and health, land and livestock, livelihood, and belonging to the vulnerable group. Adoptive capacity is defined as adaptation efficacy, self-efficacy, economic capability, human resource capability, and institutional capability. For climatic variables, respondents’ perceptions of climate change during the past ten years (2010–2019) and Pakistan meteorological data (PMD) were used in the form of the mean and standard deviation of monthly average minimum temperature, mean and standard deviation of monthly average maximum temperature, and mean and standard deviation of monthly average rainfall. Equations (1) and (3) were used to estimate $LVI_{IPCC_{V}}$ whereas Equation (4) was used to calculate the vulnerability contributing factors toward $LVI_{IPCC_{V}}$:

(4) $$CF_{v}=\frac{\sum_{i=1}^{11}W_{mi}M_{vi}}{\sum_{i=1}^{11}W_{mi}}$$

where $CF_{v}$ represents vulnerability contributing factors (exposure, sensitivity, and adoptive capacity) among the three agro-ecological zones. $M_{vi}$ denotes the major components for each zone indexed by i, and n is the number of major components in $CF_{v}$. $LVI_{IPCC_{V}}$ is calculated using Equation (5):

(5) $$LVI_{IPCC_{V}}=(CF_{e_{d}}-CF_{ac_{d}})*CF_{s_{d}}$$

where $CF_{e_{d}}$, $CF_{s_{d}}$, and $CF_{ac_{d}}$ represent factors contributing to exposure, sensitivity, and adoptive capacity for each zone, respectively. $LVI_{IPCC_{V}}$ ranges from −1 to +1 for least-vulnerable to most-vulnerable, respectively.

### 2.4. Drivers of Adoption

We analyzed drivers of adoption of climate change strategies among the sampled livestock farmers based on the information gathered about the nature of adaptation measures being followed by the farmers. Numerous local adaptation strategies were being adopted by the livestock herders. Here, we categorized four major adaptations: (1) improve feeding (diet supplements, grazing management, practicing concentrate, and bran feeding); (2) provision of medical facilities (disease control precaution, involvement in livestock training); (3) updating with seasonal and weather forecast information; and (4) livestock diversification and improved/stress-tolerant breed/species. The values of respondents adopting these strategies were 57.3%, 23.5%, 28.6%, and 16.8%, respectively. Each adaptation received a separate response relative to livestock herders’ socioeconomic characteristics. Therefore, we used a binary logit model to analyze the relationship between livestock herders’ adaptations and their socio-economic characteristics. These adaptations were considered to be a dependent variable and their values were recorded as 1 (if adopted) and 0 (otherwise). The following model was used for analysis:

$$Y_{i}=\beta_{0}+\beta_{i}X_{i}+\varepsilon_{i}$$

where $Y_{i}$ = dependent variables (adaptation strategies adopted by livestock herders), $\beta_{0}$ = constant, $\beta_{i}$ = coefficient of independent variables, $X_{i}$ = explanatory variables, and $\varepsilon_{i}$ represents the error term. The explanatory variables used in the present research were age, experience, family size, education, household type, area under fodder, farm assets, basic repair facilities in the village, off-farm income, and distance from market. Numerous studies have focused on drivers in different dimensions [11,67,68,69]. We included these variables in our study due to their anticipated impact on adaptations in numerous adoption studies [11,70,71]. Table 1 shows the anticipated signs of independent variables that were used in the study.

## 3. Results

### 3.1. Socio-Demographic Characteristics of Study Participants

Table 2 presents selected socio-demographic characteristics of the respondent livestock herders. The average age of the respondents in DGK, RYK, and FSD was 45.75, 43.61, and 43.83 years, respectively, representing middle-aged respondents. The frequency distribution of respondents’ education level showed that the majority of the respondents belonging to DGK and RYK were illiterate (53 and 42 percent, respectively) or had primary-level education (51 and 59, respectively), and had an education degree below college level. By comparison, in FSD, the education level was higher relative to the other two zones. In terms of livestock rearing experience, nearly half of the livestock herders from the three zones had more than 20 years’ experience, with average experience of 21.52, 23.32, and 21.30 years, respectively. The family size was large in all zones, i.e., around 12, 10, and 7 persons per household, respectively, of which with majority of family members were middle-aged (16–65 years).

To assess vulnerability in the context of the identification of threats from CC, resilience must also be considered in relation to the socio-economic status of the respondents, household characteristics, off farm income, and basic institutional facilities. Respondents were asked about their type of lavatory in reference to their living standard. The majority of livestock herders had flush-type lavatory systems in their homes. The results also revealed that the majority of the respondents in DGK and RYK used wood for fuel with constrained household amenities. In contrast, an opposite trend was evident in FSD where respondents used Liquified Petroleum Gas (LPG) for cooking purposes with supplemented livelihood amenities. Given the nature of the study, and because the sampled households were involved in livestock rearing, the area under fodder of the majority of the respondents was approximately equal to or less than 2 acres. The majority of the livestock herders of the three zones had to travel up to 20 km to reach different markets to sell their produce or purchase household and farm-related goods/inputs.

In the study area, respondents were also inquired about their assets for supporting their livelihood and farming operations. Here, we only considered agricultural equipment such as tractors, trolleys, tube-wells, and threshers to gain insights into respondents’ economic condition. The results in Table 2 show that the majority of the respondents owned only one or two pieces of equipment from the four machines listed above, with most having their own tube-well for irrigation purposes. The survey’s results also show that a minority of farmers had their own tractor due to poor economic conditions. However, it was previously established that ownership of agricultural assets stimulates agriculture growth and reduces poverty levels [1,75,81]. The majority of the respondents of the three zones were involved in off-farm activities for livelihood diversification. Having off-farm income sources is considered to be an adaptation measure against various risk sources, including CC, while also fostering adoptive capacity [6,74]. In addition, at the individual and household levels, larger capital endowments rapidly help to mitigate risks associated with climatic extremes. However, in developing countries such as Pakistan, smallholders remain at risk of climatic extremes due to having a poor resource base [31].

### 3.2. Livestock Herders’ Climate Change Perceptions and Meteorological Data

Study participants were asked questions regarding their concerns and perceptions about the frequency and intensity of CC events they had observed in the past 10 years. We only considered responses narrated by the majority of respondents who believed that CC was occurring in terms of climate-related events that had not previously occurred. Table 3 lists these perceptions of the respondents in the study area. It is clear that, in the past 10 years, the majority of the respondents observed an increase in high and low temperature variations. They also believed that the rainfall pattern had changed while noting a drop in the frequency of extreme climatic events (droughts and floods), and thus considered these events to be less threatening. As noted earlier, the majority of the respondents (174, 142, and 122) mentioned that the frequency of high/low temperature and rainfall intensity was high, whereas the remainder of the responses indicated the frequency was in the range of medium to very high. Respondents also observed abrupt changes in summer and winter temperatures, as being higher and lower, respectively, compared with the past. These finding agree with those of Abid et al. [37] and Ahmad and Ma [16] in the case of Punjab province. Additionally, livestock herders believed that CC led to the emergence of new diseases among their animals with an increased frequency and intensity, and indicated a frequency in the range of high/very high.

However, the measurement of climate change risk perception depends on demographic, social, economic, and cultural characteristics [70]. The perception of risk is a mental construct and personal perception may vary among individuals [82]. The literature provides numerous evidence of perceptions calculated using the Likert scale [64,65]. In the present research, we used the Likert scale to assess livestock herders’ risk perceptions regarding climate change. Table 3 shows the responses of CC risk perception events/variations over the past 10 years and the calculated values of CCRPS and SCCRPI. CCRPS values ranged from 436 to 1151 and SCCRPI values ranged from 26.914 to 71.049. The values showed that livestock herders ranked drought at the lowest level and rainfall pattern change at the highest level of risk perceived from climate change.

**Figure 3:**
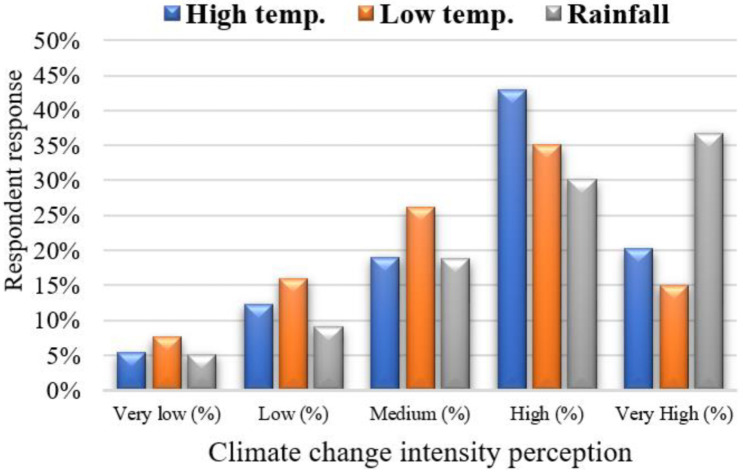
Respondents’ stated perceptions about high/low temperature and rainfall during the past 10 years.

**Figure 4:**
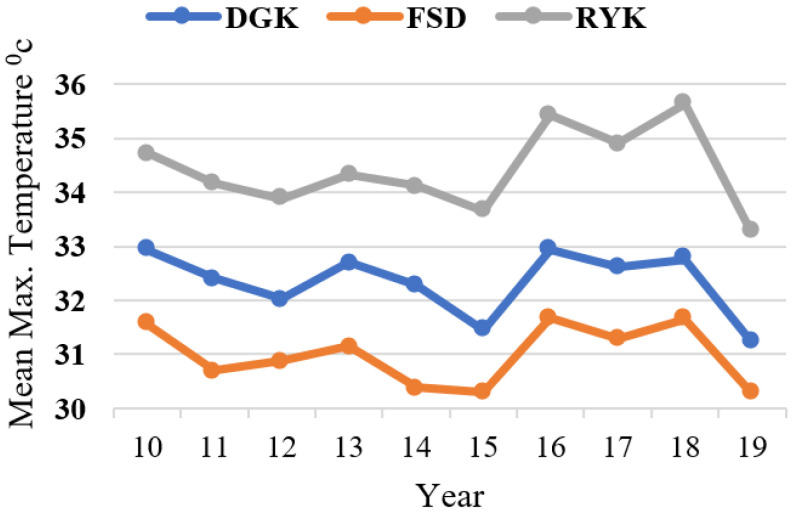
Maximum temperature (mean) of study locations (2010–2019).

**Figure 5:**
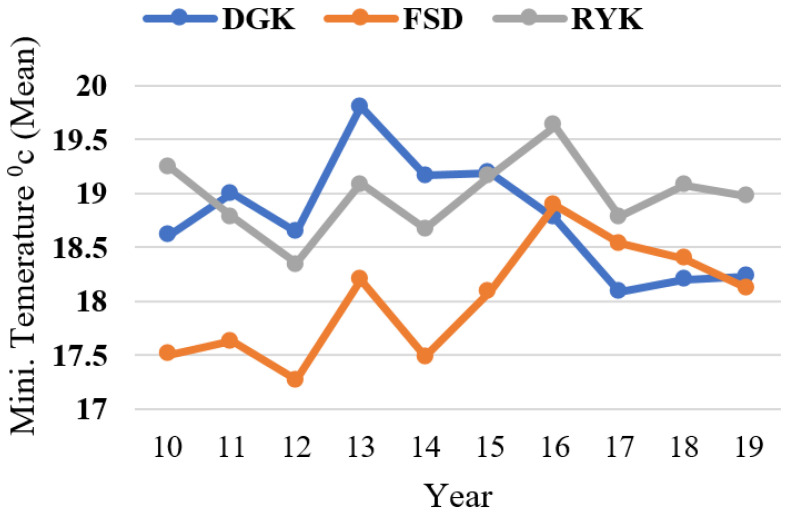
Minimum temperature (mean) of study locations (2010–2019).

**Figure 6:**
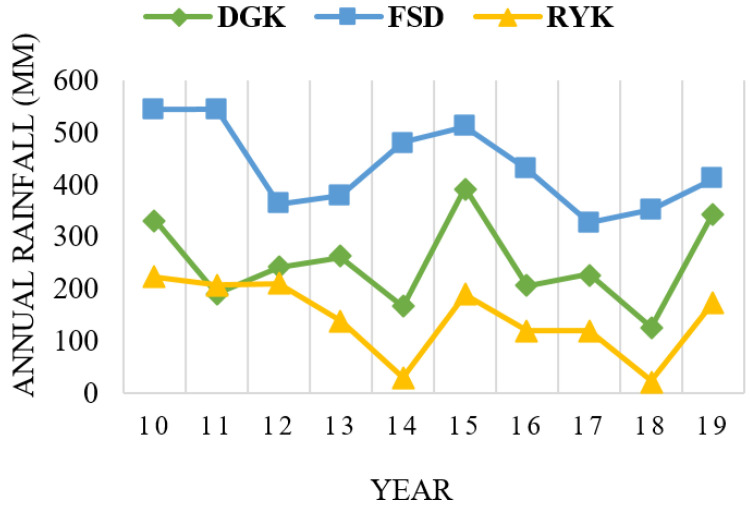
Annual rainfall in the study locations (2010–2019).

The responses relating to respondents’ perceptions about temperature (high temperature, low temperature) and rainfall pattern are shown in Figure 3. Similarly, livestock herders’ perceptions about the above-mentioned CC indicators are compared with the past 10 years’ (2010–2019) meteorological data for the study area. Results showed that respondents’ perceptions of the trends in high and low temperatures were verified by the annual mean plotted trends, as shown in Figure 4 and Figure 5, respectively. The graphs show that perceptions about temperature (high/low) were consistent with the meteorological data. The fluctuating trend in temperature is consistent with the stated perceptions of the respondents, both for summers and winters in the study locations. In the case of rainfall, the majority of respondents perceived that the pattern had also changed. The meteorological data on annual rainfall show a fluctuating trend each year during the period 2010–2019 (Figure 6), and are consistent with the respondents’ observations.

The specific climate-related characteristics of the selected agro-ecological zones for the period 2010–2019 were derived from the processing of meteorological data. The results show that, from 2010 to 2019, the annual minimum/maximum temperature (mean) of DGK, RYK, and FSD were 18.77/32.34, 18.98/34.41, and 18.01/30.99, respectively. The annual rainfall from 2010 to 2019 of DGK, RYK, and FSD was 248.98, 143.43, and 434 mm, respectively. Consequently, these zones were characterized by a mean annual temperature in the range of around 18.6–32.6 °C, and total annual rainfall in the range of 143 to 434 mm, during the study period.

### 3.3. Contributing Factors of Vulnerability

#### 3.3.1. Exposure Assessment

Exposure is considered to be a major dimension of vulnerability, and refers to changes in key variables of the climatic system (e.g., precipitation and temperature) and extreme events (drought, flood, and animal diseases). In the present research, the exposure assessment was based on respondents’ perceptions and was compared with the regional climatic data (rainfall, minimum temperature, maximum temperature) provided by the Pakistan meteorological department (PMD). Within exposure, two major components were categorized into nine sub-components (Table 4): extreme events (defined as past 10 years’ experienced animal diseases, drought, and flood intensity), and climatic variables (defined as past 10 years’ observed min/max temperature variation, rainfall and region-wise PMD data of annual mean min/max temperature and rainfall).

The analysis showed that livestock herders from DGK were more exposed to extreme events and more vulnerable to drought (0.535), flood (0.543), and animal diseases (0.774) compared with those in RYK and FSD, who had relatively milder exposure to drought (0.141, 0.131), flood (0.128, 0.285), and animal diseases (0.561, 0.689), respectively. The average scores of the extreme events were 0.617, 0.227, and 0.369 for DGK, RYK, and FSD, respectively, signifying a greater exposure of DGK’s livestock herders to extreme events than those in the other two zones. Similarly, district-wise average scores of climatic variables were 0.584, 0.548, and 0.607 for DGK, RYK, and FSD, respectively, implying a greater exposure of FSD to climatic stimuli than the other two zones (Table 5).

#### 3.3.2. Sensitivity Assessment

In the assessment of vulnerability, sensitivity to CC was estimated on the basis of four major components: food and health, land and livestock, livelihood, and belonging to the vulnerable group. These four major components were further sub-divided into several sub-components, as reported in Table 4. The major components of food and health were measured by the response to six sub-components defined as the past ten years’ observation/trend/consumption, such as the depth of subsoil water, dairy yields and/or milk production per family, milk in diet, meat in diet, child growth performance, and amount of food consumed. The land and livestock component was accounted for by the average landholding per household, past ten years’ livestock losses, fodder shortage, and change in the quantity of livestock. The livelihood component questioned whether, in the past ten years, respondents removed their children from school, changed their employment/work pattern, or applied for an extended loan term due to climatic disaster(s). The vulnerable group was defined as family members who were below 15 or above 65 years of age. The results in Table 5 indicate that respondents were more sensitive to food and health (0.503) in DGK, land and livestock (0.356) in FSD, and livelihood and the vulnerable group (0.489, 230, respectively) in RYK. The overall score of the sensitivity index indicates that RYK was more sensitive (0.395) in comparison with DGK and FSD (0.375 and 0.328, respectively) (see Table 5).

#### 3.3.3. Adaptive Capacity Assessment

Adoptive capacity assessment was undertaken on the basis of following major components: adaptation efficacy, self-efficacy, economic capability, human resource capability, and institutional capability, as illustrated in Table 4. Adaptation and self-efficacy were measured on a five-point scale—1 (strongly disagree) to 5 (strongly agree). Economic capability assessed respondents’ financial and structural barriers and total number of livestock. Human resource capability was assessed by taking into account the household head’s education, livestock rearing experience, and adult family members (number). Institutional capability was assessed on the basis of the distance of the household’s residence from main/link road and market, and availability of basic facilities in the village. The scores of adaptation efficacy (0.559, 0.524, and 0.480), self-efficacy (0.456, 0.621, and 0.539), economic capability (0.384, 0.391, and 0.345), human resource capability (0.302, 0.314, and 0.475), and institutional capability (0.277, 0.351, and 0.477), estimated for DGK, RYK, and FSD, respectively, show a mixed picture. The overall district-level scores of the adaptive capacity index for DGK, RYK, and FSD zones, respectively, were 0.375, 0.422, and 0.465, reflecting a low level of adaptive capacity for DGK and RYK to cope with CC (Table 5). Therefore, southern Punjab (DGK, RYK) livestock herders had low adaptive capacity due to a low level of education, less-developed infrastructure, and poor household facilities, as noted during the field survey.

### 3.4. Vulnerability Index Assessment

**Figure 7:**
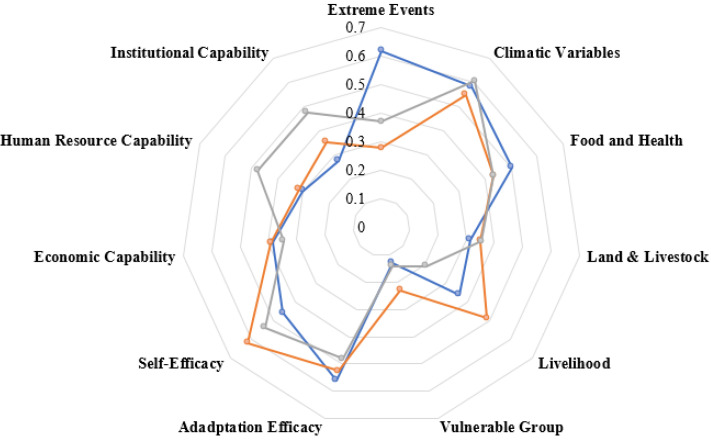
Spider diagram of vulnerability based on major components of LVI of the study area.

Based on the findings related to the contributing factors of vulnerability, DGK was the most-vulnerable of the three districts, followed by FSD and RYK. The spider diagram of vulnerability in Figure 7 represents the LVI values encompassing all 11 major components calculated from 36 sub-components (see Table 5 for sub-component results). LVI is scaled from 0 (least vulnerable) to 0.7 (most vulnerable). All sub-component index values and LVI outcomes based on the former are shown in Table 5. Overall, LVI outcomes indicate that DGK (LVI = 0.4309) was more vulnerable than FSD (LVI = 0.4237) and RYK (LVI = 0.4198). DGK was more vulnerable in terms of extreme events (0.617), food and health (0.503), self-efficacy (0.456), human resource capability (0.302), and institutional capability (0.277); FSD was more vulnerable in terms of climatic variables (0.607), land and livestock (0.356), and economic capability (0.345); and RYK was more vulnerable in terms of livelihood (0.489) and the vulnerable group category (0.230) (Table 5 and Figure 7).

**Figure 8:**
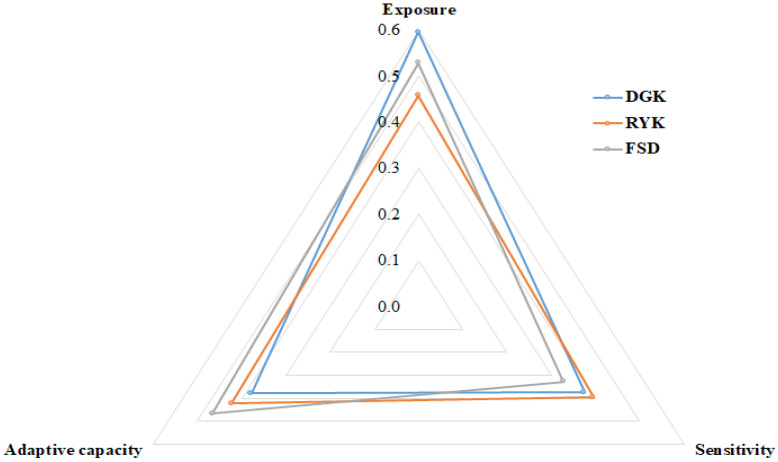
LVI-IPCC contributing factors of the three agro-ecological zones.

The LVI-IPCC scale ranged from −1 (less-vulnerable) to 1 (most-vulnerable). Small livestock herders in the DGK were vulnerable to CC in terms of exposure (0.595) with lower adaptive capacity (0.378). Moreover, respondents in FSD were also vulnerable although less exposed (0.528), having lower sensitivity to CC (0.328) and a higher level of adaptive capacity (0.465) compared with DGK. The farmers in RYK were the least vulnerable, despite being more sensitive (0.395) to CC with a lower level of exposure (0.458) and a higher level of adaptive capacity (0.422) compared with the other two districts (Table 6 and Figure 8).

In total, DGK was the most vulnerable district of the three (Table 6). The results imply that the high vulnerability level in DGK is attributable to lower adaptive capacity, higher sensitivity, and higher exposure to CC. The higher vulnerability in DGK is due to different factors, such as the farmers’ increased dependency on livestock and widespread poverty in the region [16]. Nonetheless, the increase in the frequency and intensity of floods, droughts, and the incidence of new diseases among livestock have marred livestock production. As a result, farmers are more vulnerable with respect to food, health, and livelihood sustenance. The other reasons for increased vulnerability are subsistence livelihood options, such as small farm sizes, poor self-efficacy, and low economic capability, leading to decreased livestock production and lower farm revenues [70]. Moreover, the least involvement in off-farm income generation activities in this zone, compared with the RYK and FSD zones, further intensify the region’s vulnerability to CC. Human resource capability and institutional capability in DGK were also lower compared to those of the other zones. These factors render DGK inhabitants highly vulnerable, severely exposed, and physically and structurally sensitive. As a result, these farmers have poor adaptive capacity because small impacts on these livestock enterprises disturb the existing balance of overall households’ welfare within the mix of available resources [16].

### 3.5. Drivers Influencing Herders’ Adaptations

Table 7 shows the results of drivers influencing livestock herders’ adaptations to climate change. The results show that the coefficient of family size is positive and highly significant, which indicates that livestock herders with larger family size adopt more adaptations because of the accessibility of manpower required to manage the livestock. In addition, the coefficient of education is highly significant and positive, which emphasizes that educated livestock herders are likely to adopt more adaptations. The coefficient of household type is significant and positive, which indicates that households living in an extended/joint family type are likely to adopt more adaptations. The reason for this also relates to the family size; joint families have excess labor who are available to look after their livestock. The coefficient of cooking fuel is negative and only significant for the third adaptation. The overall negative sign indicates that smallholders have limited resources and suffer from financial constraints, and that if they used LPG as a cooking fuel, they did not have sufficient resources to spend on adaptations. The coefficient of off-farm work is negative and significant, indicating that off-farm work lessens the time allocation for livestock maintenance. Age, area under fodder, farm assets, and distance to market are non-significant, implying that these do not affect respondents’ adoption behavior regarding climate change mitigation strategies.

## 4. Discussion

The majority of livestock herders perceived climate variability during the last ten years to be a major stimulus of increased vulnerability in the study area. More than 75% of respondents perceived a medium to high level of variations/patterns within the study district. This is expected because residents previously reported that numerous parts of the province experienced such impacts [16,37]. In the comparison of individuals’ perceptions with the actual meteorological data of the recent past, to confirm if the former were supported by evidence, we found a genuine link between the former and the latter [16]. Respondents also reported the need for equipment and financial/technical support to help in adopting climate-smart practices to achieve sustained farm production, and to secure and diversify their livelihoods. In the study locations, CC mitigation measures were inadequate due to widespread poverty among respondents, who largely depended on livestock for subsistence. This inadequacy was exacerbated by lower education status, a poor resource base, and constrained institutional support. Given these factors, outside support from NGOs, and public and private sectors, is necessary for the implementation of effective adaptations, which will achieve and demonstrate benefits for poorer areas or those living in the vicinity [34,70,83]. The limited adaptation ability of rural households may be further complicated by the multiplier effect due to the declining productivity per unit of land. This results in a problem of food insecurity for the households themselves and urban consumers, thus leading to a decline in health status. In turn, this places significant pressure on foreign exchange to fulfil domestic demand via imports [84]. This circle will continue until households are capable of improving their self-efficacy to adopt CC measures [70]. As reported by Rivera-Ferre et al. [85], adoption of new strategies can improve production and food availability.

The previous literature has noted that rural people who are associated with the farming sector are severely affected by the negative impact of CC [86,87]. Ahmad and Ma [16] reported similar findings, which indicated that a decrease in precipitation, longer summers, and variations in the growing season were verified by farming communities. The magnitude and frequency of climatic extremes such as floods, droughts, and temperature fluctuations have been anticipated and are realized in recent years [59,88]. Numerous researchers have argued that environmental factors are not only responsible for vulnerability, but also the poverty levels of countries [41,52,89,90]. Moreover, the majority of the populations in developing countries rely on small-scale livelihoods, and have lower adaptive capacity, which creates significant challenges in coping with CC [5]. Large populations of developing countries, including Pakistan, are poor and live in rural, disaster-prone areas. Strengthening institutions and providing economic support are the mast-effective means to mitigate CC impacts under these scenarios.

The current investigation revealed that livestock herders in the three study zones observed regular shocks to their livelihood due to livestock diseases and deaths caused by extreme climatic events. In particular, they were often faced with low livestock productivity due to fodder shortages, herd size reductions, or livestock losses. They also experienced food insecurity due to low crop and livestock yields. The strategies adopted by the livestock herders were based on the reduction in livestock products’ consumption. These results are in line with previous studies, in which significant impacts of CC on the livelihood of smallholders are reported [5,8]. The generation of additional income by engaging in off-farm activities or changing employment/work patterns is reported to be an effective response. These adaptive measures evidently help to moderate the negative impacts on livestock herders; however, this approach is considered unsatisfactory in conditions of severe insecurity [14]. Additionally, adaptive measures to earn additional income are considered insufficient due to inadequate opportunities for off-farm wage laborers due to their poor skill and knowledge capital [12,74]. Other reasons, such as poor infrastructure and lack of institutional services, may also impact households’ motivation to engage in alternate earning opportunities [6]. Significant efforts are required to improve the livelihood of small livestock herders, with a special focus on increasing livestock productivity and reducing the vulnerability of their livelihoods to climate-related risks through a variety of other interventions. These interventions may include the provision of improved or stress-tolerant breeds and species, improving the institutional capability (infrastructure, markets access, and basic facilities), and the provision of human resource capability (technical education and expertise). The results indicated that the provision of veterinary services to enhance technical skills in these vulnerable zones, and the promotion of training programs related to the best management practices for the adoption of new technology, helped to increase livestock productivity and reduce vulnerability. The presence of functional facilities—for example, social safety nets and access to credit during catastrophes—and education in new techniques, can provide the ability to mitigate risks and maintain livestock productivity. Appropriate policies and programs are required to provide alternative measures for livelihood support and livestock diversification to reduce farmers’ vulnerability. Omerkhil et al. [8] and Jha et al. [91] stated that properly developed government-sponsored rural development programs have improved welfare overall because these programs further support the ability of smallholders to increase their resilience to negative impacts of climate change.

## 5. Conclusions

The present research evaluated the livelihood vulnerability (LVI, LVI_IPCC_) of small livestock herders of three agro-ecological zones of Punjab, Pakistan. The LVI is a suitable method for the assessment of critical factors in which equal weight is applied to all major components and sub-components, and provides a better means of comparing the indicators across different regions at the household level. Moreover, this approach can help policy makers to identify the most vulnerable zones, and to develop response policies for the allocation of maximum resources in areas that are prone to the challenges associated with climate change.

Based on the results, this study offers the following specific policy recommendations. First, in the DGK zone, the priority is to focus on food and health, human resource capability, and institutional capability. Second, FSD requires timely information regarding climatic variations and disease control precautions to reduce livestock losses, because it is expected that the livestock sector may grow more quickly than crop farming in the future. Third, RYK requires financial support, and technical and professional assistance, to curb climate vulnerability. Poor food and health conditions, sensitive livelihood conditions, and lower economic, institutional, and human resource capabilities, are the prime reasons for small livestock herders’ vulnerability in the study region. The main risks are a high frequency of disease outbreaks, variations in rainfall patterns, and temperature changes due to CC. Therefore, it is vital to reduce the current and future livelihood vulnerability to climate-related risks of smallholder livestock herders by increasing their productivity and resilience to CC. This requires a small number of low-cost and local approaches, such as improving veterinary services, reinforcing informal social safety nets, and applying small-scale local infrastructure projects. These approaches may represent feasible, cost-effective, and sustainable decisions, and encourage the development of a mindset of low periodic costs and the minimum maintenance required. The vulnerability results show that a large number of small livestock herders who are vulnerable to CC need educational, economic, and institutional support to improve their coping capacity. The assessment of critical indicators identified more specific future policy directions to combat livelihood vulnerability. From the assessed indicators, the policy targets comprise food and health projects, awareness of climatic vulnerabilities, and institutional capabilities for under-developed zones. Therefore, it is crucial to increase the adaptive capacity of small villages at the local scale to increase the resiliency of smallholders to combat the global threat of CC.

### Study limitations

Different approaches can be used to measure vulnerability. In the present study, an index-based method was used to evaluate vulnerability. Although this is a practical approach to explore the conceptual framework and to monitor different trends, it has certain limitations, as follows. (1) Due to the analytical approach, we were confined in the selection of variables, authentication of various measurement units, and calculation of relative weights. We did not include all of the components that may affect the vulnerability of the region because the necessary improvement required the construction of major and minor components of vulnerability to enable a comprehensive evaluation. (2) Respondents are better able to recall recent trends in atmospheric condition, rather than earlier changes during the past decade, and CC is a long-term phenomenon. (3) Multidimensional data to evaluate vulnerability was lacking because we were confined to the assessment of government-provided indicators only.

## Figures and Tables

**Table 1:** Description of model variables.

Explanatory Variables	Expected Sign	Reference
Age (years)	−,+	[71,72]
Experience (Years)	+	[63,70,73,74,75]
Family Size (Years)	+	[34,76]
Education	+	[74,75,77,78]
Household type	+	[11,34,76]
Area under fodder (Acre)	−,+	[11,34,70]
Farm Assets (number)	+	[79]
Cooking fuel	−,+	[80]
Basic repair facilities in village	+	[70]
Off-farm income	−,+	[70,74]
Distance to market (km)	−,+	[70]

**Table 2:** Socio-demographic and economic characteristics of study participants.

Characteristics	Group	Agro-Ecological Zones (Study Area)		
		DGK	RYK	FSD
Age (years)	≤30	11	17	17
	31–50	82	89	86
	≥50	42	29	32
Experience (Years)	≤20	63	61	74
	21–35	65	56	52
	≥36	7	18	9
Age-wise Avg. Number of Family Members (Years) *	≤15	4.71	3.96	2.44
	15 ≤ age ≤ 65	7.03	6.04	4.56
	≥65	0.41	0.26	0.42
	Total	12.15	10.26	7.42
Education	Illiterate	53	42	21
	Primary	51	59	19
	High school	23	29	55
	College/above	8	5	40
Household type	Nuclear	60	57	33
	Joint	75	78	102
Area under fodder (Acre)	≤2 acre	109	130	117
	≥2.1 acre	26	5	18
Farm Assets (number)	Zero asset	38	55	42
	1–2	72	58	48
	3–4	25	22	45
Cooking fuel	Wood	126	98	35
	LPG (and others)	9	37	100
Basic repair facilities in village	No	95	92	31
	Yes	40	43	104
Off-farm income	No	62	27	64
	Yes	73	108	71
Distance to market (km)	≤10	48	64	8
	11–20	70	46	70
	≥21	17	25	57

Source: Field survey. Note: All the variables in this table report frequencies, with the exception of family size, which is represented by the average number of family members in various age categories. * This information is based on the average number of family members per household for the three age-groups, i.e., ≤15 years, 15–65 years, and above 65 years of age. Summing the mean number of family members in each age-category yields the average household size for respective agro-ecological zone.

**Table 3:** Responses of CC risk perception events/variations during the past 10 years that rarely occurred previously.

Climate Change Events	Frequency					CCRPS	SCCRPI	Rank
	Very Low	Low	Medium	High	Very High			
Drought	184	95	67	29	30	436	26.914	6
High temperature	22	50	77	174	82	1054	65.062	3
Low temperature	31	65	106	142	61	947	58.457	4
Animal diseases	27	47	81	116	134	1093	67.469	2
Rainfall Pattern Change	21	37	76	122	149	1151	71.049	1
Flood	186	96	47	55	21	439	27.099	5

Source: Field survey.

**Table 4:** Vulnerability indicators (major components and sub-components) and functional relationship with vulnerability.

Contributing Factors	Major Components	Sub Components (Indicators)	Description	Relationship **
**Exposure**	Extreme Events	Past 10 years observed drought intensity	Measured in a 5 point scale 1 (very low) to 5 (very high)	+
		Past 10 years observed flood intensity	Measured in a 5 point scale 1 (very low) to 5 (very high)	+
		Past 10 years observed animal diseases	Measured in a 5 point scale 1 (very low) to 5 (very high)	+
	Climatic Variables	Past 10 years observed high temperature variation	Measured in a 5 point scale 1 (very low) to 5 (very high)	+
		Past 10 years observed low temperature variation	Measured in a 5 point scale 1 (very low) to 5 (very high)	+
		Past 10 years observed rainfall variation	Measured in a 5 point scale 1 (very low) to 5 (very high)	+
		Annual mean minimum temperature °C (2010–2019) PMD *	Mean standard deviation of monthly average minimum temperature	+
		Annual mean maximum temperature °C (2010–2019) PMD *	Mean standard deviation of monthly average maximum temperature	+
		Annual mean rainfall (2010–2019) PMD *	Mean standard deviation of monthly average rainfall	-
**Sensitivity**	Food and Health	Increase in the depth of subsoil water (past 10 years observation)	Percentage	+
		Dairy yields/milk production/family (past 10 years trend)	Measured in a 3 point scale (1) no change (2) decrease (3) increase	+
		Milk in diet (respondent past 10 years consumption trend)	Measured in a 3 point scale (1) no change (2) decrease (3) increase	+
		Meat in diet (respondent past 10 years consumption trend)	Measured in a 3 point scale (1) no change (2) decrease (3) increase	+
		Child growth performance (respondent past 10 years observation)	Measured in a 3 point scale (1) no change (2) decrease (3) increase	+
		Amount of food consumed was below than desired quantity (respondent past 10 years observation)	Measured in a 3 point scale (1) No (2) yes for a couple a day’s (3) yes for a couple of weeks	+
	Land and Livestock	Average land of household members (acre)	Own land/total number of family members	-
		Number of livestock losses in past 10 years (count)	Number	+
		Have you experienced fodder shortage in past 10 years?	(1) Yes (0) otherwise	+
		Change in total number of livestock during past 10 years	Measured in a 3 point scale (1) no change (2) decrease (3) increase	+
	Livelihood	Took out children from school in past 10 years	(1) Yes (0) otherwise	+
		Have you changed the employment or work pattern in past 10 years	(1) Yes (0) otherwise	+
		Applied for extended term of loan due to climate disaster in past 10 years	(1) Yes (0) otherwise	+
	Vulnerable Group	Household members less than 15 years (count)	Number	+
		Household members greater than 65 years (count)	Number	+
**Adaptive Capacity**	Adaptation Efficacy	I am very positive about climate change adoption measures	Measured in a 5 point scale 1 (strongly disagree) to 5 (strongly agree)	-
		I plan to adopt measures for climate change	Measured in a 5 point scale 1 (strongly disagree) to 5 (strongly agree)	-
	Self-Efficacy	It is mostly up to me, whether or not to adopt climate change measures for my livestock	Measured in a 5 point scale 1 (strongly disagree) to 5 (strongly agree)	±
		I have adequate ability (knowledge and skills) to implement climate change measures on my farm	Measured in a 5 point scale 1 (strongly disagree) to 5 (strongly agree)	-
	Economic Capability	Financial and structural barrier prohibit me to adopt climate change measures	Measured in a 5 point scale 1 (strongly disagree) to 5 (strongly agree)	+
		Total number of livestock (count)	Number	-
	Human Resource Capability	Adult family members (count)	Number	-
		Household head education (years)	Years	-
		Livestock experience (years)	Years	-
	Institutional Capability	Distance to reach the road (km)	Km	+
		Distance to market (km)	Km	+
		Basic repair facilities available in village	(1) Yes (0) otherwise	-

* Pakistan meteorological department; ** Relationship between vulnerability and indicator: (+) represents positive relationship between vulnerability and indicator, and (−) represents negative.

**Table 5:** Indexed sub-components and overall livelihood vulnerability index (LVI).

Major-Components	Code	Sub-Components	Agro-Ecological Zones		
			DGK	RYK	FSD
Extreme Events	EXP1	Past 10 years observed drought intensity	0.535	0.141	0.131
	EXP2	Past 10 years observed flood intensity	0.543	0.128	0.285
	EXP3	Past 10 years observed animal diseases	0.774	0.561	0.689
			**0.617**	**0.277**	**0.369**
Climatic Variables	EXP4	Past 10 years observed high temperature variation	0.556	0.526	0.648
	EXP5	Past 10 years observed low temperature variation	0.594	0.472	0.687
	EXP6	Past 10 years observed rainfall variation	0.758	0.578	0.735
	EXP7	Annually mean standard deviation of minimum temperature (2010–2019) PMD *	0.538	0.352	0.526
	EXP8	Annually mean standard deviation of max temperature (2010–2019) PMD *	0.599	0.762	0.557
	EXP9	Annual rainfall (2010–2019) PMD *	0.461	0.600	0.490
			**0.584**	**0.548**	**0.607**
Food and Health	SEN1	Increase in the depth of subsoil water (past 10 years observation)	0.493	0.410	0.293
	SEN2	Dairy yields/milk production/family (past 10 years trend)	0.541	0.456	0.470
	SEN3	Milk in diet (respondent past 10 years consumption trend)	0.581	0.441	0.452
	SEN4	Meat in diet (respondent past 10 years consumption trend)	0.515	0.441	0.433
	SEN5	Child growth performance (respondent past 10 years observation)	0.407	0.370	0.415
	SEN6	Amount of food consumed was below than desired quantity (respondent past 10 years observation)	0.481	0.481	0.526
			**0.503**	**0.433**	**0.432**
Land and Livestock	SEN7	Average land of household members	0.078	0.150	0.160
	SEN8	Number of livestock losses in past 10 years	0.291	0.356	0.363
	SEN9	Have you experienced fodder shortage in past 10 years?	0.541	0.607	0.415
	SEN10	Change in total number of livestock past 10 years	0.344	0.289	0.485
			**0.314**	**0.350**	**0.356**
Livelihood	SEN11	Took out children from school in past 10 years	0.370	0.496	0.207
	SEN12	Have you changed the employment or work pattern in past 10 years	0.519	0.519	0.244
	SEN13	Applied for extended term of loan due to climate disaster in past 10 years	0.193	0.452	0.170
			**0.360**	**0.489**	**0.207**
Vulnerable Group	SEN14	Household members less than 15 years	0.128	0.330	0.144
	SEN15	Household members greater than 65 years	0.138	0.130	0.141
			**0.133**	**0.230**	**0.142**
Adaptation Efficacy	AC1	I am very positive about climate change adoption measures	0.591	0.594	0.541
	AC2	I plan to adopt measures for climate change	0.528	0.454	0.419
			**0.559**	**0.524**	**0.480**
Self-Efficacy	AC3	It is mostly up to me, whether or not to adopt climate change measures for my livestock	0.493	0.685	0.596
	AC4	I have adequate ability (knowledge and skills) to implement climate change measures on my farm	0.420	0.557	0.481
			**0.456**	**0.621**	**0.539**
Economic Capability	AC5	Financial and structural barrier prohibit me to adopt climate change measures	0.520	0.591	0.619
	AC6	Total number of livestock	0.247	0.190	0.071
			**0.384**	**0.391**	**0.345**
Human Resource Capability	AC7	Adult family members	0.148	0.311	0.414
	AC8	Household head education	0.294	0.281	0.565
	AC9	Livestock experience	0.465	0.351	0.446
			**0.302**	**0.314**	**0.475**
Institutional Capability	AC10	Distance to reach the road	0.231	0.357	0.233
	AC11	Distance to market	0.304	0.378	0.428
	AC12	Basic repair facilities available in village	0.296	0.319	0.770
			**0.277**	**0.351**	**0.477**
Overall livelihood vulnerability index (LVI) *			0.4309	0.4198	0.4237

* Note: LVI scale→0 (least vulnerable) to 0.7 (most vulnerable).

**Table 6:** Table LVI-IPCC contributing factors of three agro-ecological zones.

Contributing Factors	DGK	RYK	FSD
**Exposure**	0.595	0.458	0.528
**Sensitivity**	0.375	0.395	0.328
**Adaptive capacity**	0.378	0.422	0.465
LVI-IPCC *	0.081	0.014	0.020

* LVI-IPCC scale = −1 (less vulnerable) to 1 (most vulnerable).

**Table 7:** Drivers influencing livestock herders’ adaptations to climate change.

Explanatory Variables	Response Variables			
	Model 1	Model 2	Model 3	Model 4
Age (years)	−0.012 (0.017)	−0.031 (0.025)	−0.016 (0.023)	−0.035 (0.028)
Experience (Years)	0.040 ** (0.017)	0.038 (0.026)	0.033 (0.024)	0.035 (0.029)
Family Size (Years)	0.070 ** (0.027)	0.051 ** (0.020)	0.059 *** (0.021)	0.059 *** (0.020)
Education	0.100 *** (0.033)	0.306 *** (0.047)	0.301 *** (0.045)	0.165 *** (0.047)
Household type	0.711 *** (0.245)	1.076 *** (0.328)	1.127 *** (0.326)	0.606 * (0.364)
Area under fodder (Acre)	0.143 (0.130)	0.023 (0.129)	−0.025 (0.122)	0.004 (0.121)
Farm Assets (number)	0.106 (0.130)	−0.158 (0.160)	0.020 (0.145)	0.197 (0.153)
Cooking fuel	−0.230 (0.346)	−0.446 (0.444)	−0.713 * (0.413)	−0.090 (0.462)
Basic repair facilities	0.610 ** (0.275)	0.307 (0.359)	1.199 *** (0.349)	1.246 *** (0.426)
Off-farm income	−0.532 * (0.287)	−1.101 *** (0.352)	−1.041 *** (0.343)	−1.169 *** (0.380)
Distance to market (km)	0.013 (0.018)	−0.001 (0.022)	0.018 (0.021)	0.028 (0.024)
DGK	0.503 (0.392)	1.931 *** (0.537)	0.385 (0.478)	0.424 (0.572)
RYK	0.585 (0.380)	1.751 *** (0.499)	0.633 (0.449)	1.171 ** (0.525)
FSD	Omitted	Omitted	Omitted	Omitted
Constant	−2.139 ** (0.882)	−3.932 *** (1.092)	−3.956 *** (1.065)	−4.270 *** (1.230)
Observations	405	405	405	405
Pseudo R2	0.143	0.251	0.295	0.244
Log Likelihood	−236.623	−165.155	−170.806	−138.495
Prob > chi2	0.0000	0.0000	0.0000	0.0000

Note: S.E reported in parentheses; ***, **, *, are significant at the *p* < 0.01, *p* < 0.05, *p* < 0.10 level, respectively.

## Data Availability

Data may compromise the privacy of study participants and may not be shared publicly. Data are available upon request to the Xia Chunping, Professor in College of Economics and Management, Huazhong Agricultural University, Wuhan, China Email: xcp@mail.hzau.edu.cn.
